# Genome Sequences of *Gordonia* Phages Bowser and Schwabeltier

**DOI:** 10.1128/genomeA.00596-16

**Published:** 2016-08-11

**Authors:** Matthew T. Montgomery, Welkin H. Pope, Zachary M. Arnold, Aleksandra Basina, Ankitha M. Iyer, Ty H. Stoner, Naomi S. Kasturiarachi, Catherine A. Pressimone, Johnathon G. Schiebel, Emily C. Furbee, Sarah R. Grubb, Marcie H. Warner, Rebecca A. Garlena, Daniel A. Russell, Deborah Jacobs-Sera, Graham F. Hatfull

**Affiliations:** Department of Biological Sciences, University of Pittsburgh, Pittsburgh, Pennsylvania, USA

## Abstract

*Gordonia* phages Bowser and Schwabeltier are newly isolated phages infecting *Gordonia terrae* 3612. Bowser and Schwabeltier have similar siphoviral morphologies and their genomes are related to each other, but not to other phages. Their lysis cassettes are atypically situated among virion tail genes, and Bowser encodes two tyrosine integrases.

## GENOME ANNOUNCEMENT

*Gordonia* spp. are implicated in foaming of activated sludge in wastewater treatment as well as in opportunistic infections of catheters (1–4). Previously, 17 bacteriophages of *Gordonia* have been isolated, sequenced, and annotated (5–9). Isolation and genomic analysis of bacteriophages using *Gordonia terrae* 3612 as a host within the Science Education Alliance-Phage Hunters Advancing Genomics and Evolutionary Science (SEA-PHAGES) will expand our understanding of the genetic diversity of these viruses (10).

*Gordonia* phages Bowser and Schwabeltier were isolated by direct plating of filtered soil extracts from Pittsburgh, PA on *G. terrae* 3612. Following plaque purification and amplification, DNA was extracted and sequenced using an Illumina MiSeq with 140 bp single-end reads. Reads were assembled using Newbler into single major contigs of 46,570 bp and 46,895 bp for Bowser and Schwabeltier, respectively, with 1,945-fold and 1,555-fold coverage. The genomes are 67% G+C and have discrete ends with 10 base 3′ single stranded extensions (5′-CGCCGCGGTA). Bowser and Schwabeltier share segments of similarity spanning 60% of their genome lengths with nucleotide identity ranging from 82% to 94%, and are grouped together in Cluster DB using previously described parameters (11). Bowser and Schwabeltier are not closely related to other phages or prophages, although a 1.3 kbp segment is related (75% nucleotide identity) to minor tail protein genes of a putative prophage in *Gordonia* sp. KTR9 (12).

Using GeneMark (13), Glimmer (14), Phamerator (15), and DNA Master (http://cobamide2.bio.pitt.edu), we identified 71 and 72 protein encoding genes in Bowser and Schwabeltier, respectively, approximately 40% of which we could assign putative functions using BLAST (16) and HHpred (17, 18). Neither genome contains tRNA genes. Protein functional assignments include virion structure and assembly proteins, tyrosine integrases, immunity repressors, FtsK-like proteins, an acetyltransferase (Schwabeltier gp30), and several HNH endonucleases.

The lysis cassette in Bowser and Schwabeltier is unusually located within the minor tail protein genes, and includes endolysin and lysin B genes flanking four smaller open reading frames, three of which (e.g., Schwabeliter gp22, gp23, and gp25) are strongly predicted to be membrane proteins. The three putative membrane proteins may all be associated with lysis although it is unclear which plays the holin role. Both phages have leftwards-transcribed tyrosine integrase and immunity repressor genes near the centers of their genomes, and these have the characteristics of the previously described integration-dependent immunity systems (19) in that both the integrases and repressors have C-terminal protein degradation tags. Although the *attP* site is expected to be located within the repressor-coding region in these systems, BLASTn searches failed to identify a corresponding *attB* site in any sequenced *Gordonia* genome. Curiously, Bowser encodes a second rightwards-transcribed tyrosine integrase, but we have also been unable to identify its corresponding *attP* site. It thus remains unclear whether either Bowser or Schwabeltier form lysogens with integrated prophages in *G. terrae* 3612 or any other bacterial host.

### Accession number(s).

The Bowser and Schwabeltier genomes are available from GenBank under accession numbers KU998235 and KU963252.
